# Twitter Strategies for Web-Based Surveying: Descriptive Analysis From the International Concussion Study

**DOI:** 10.2196/resprot.4542

**Published:** 2016-09-01

**Authors:** Sharief Hendricks, Peter Düking, Stephen D Mellalieu

**Affiliations:** ^1^ Division of Exercise Science and Sports Medicine Department of Human Biology University of Cape Town Cape Town South Africa; ^2^ Integrative and Experimental Training Science Institute for Sport Sciences Julius-Maximilians University Würzburg Würzburg Germany; ^3^ Cardiff School of Sport College of Engineering Cardiff Metropolitan University Cardiff United Kingdom

**Keywords:** Twitter, social media, Web-based surveying, concussion, rugby

## Abstract

**Background:**

Social media provides researchers with an efficient means to reach and engage with a large and diverse audience. Twitter allows for the virtual social interaction among a network of users that enables researchers to recruit and administer surveys using snowball sampling. Although using Twitter to administer surveys for research is not new, strategies to improve response rates are yet to be reported.

**Objective:**

To compare the potential and actual reach of 2 Twitter accounts that administered a Web-based concussion survey to rugby players and trainers using 2 distinct Twitter-targeting strategies. Furthermore, the study sought to determine the likelihood of receiving a retweet based on the time of the day and day of the week of posting.

**Methods:**

A survey based on previous concussion research was exported to a Web-based survey website Survey Monkey. The survey comprised 2 questionnaires, one for players, and one for those involved in the game (eg, coaches and athletic trainers). The Web-based survey was administered using 2 existing Twitter accounts, with each account executing a distinct targeting strategy. A list of potential Twitter accounts to target was drawn up, together with a list of predesigned tweets. The list of accounts to target was divided into ‘High-Profile’ and ‘Low-Profile’, based on each accounts’ position to attract publicity with a high social interaction potential. The potential reach (number of followers of the targeted account), and actual reach (number of retweets received by each post) between the 2 strategies were compared. The number of retweets received by each account was further analyzed to understand when the most likely time of day, and day of the week, a retweet would be received.

**Results:**

The number of retweets received by a Twitter account decreased by 72% when using the ‘high-profile strategy’ compared with the ‘low-profile strategy’ (incidence rate ratio (IRR); 0.28, 95% confidence interval (CI) 0.21-0.37, *P*<.001). When taking into account strategy and day of the week, the IRR for the number of retweets received during the hours of 12 AM to 5:59 AM (IRR 2.98, 95% CI 1.88-4.71, *P*>.001) and 6 PM to 11:59 PM (IRR 1.48, 95% CI 1.05-2.09, *P*>.05) were significantly increased relative to 6 AM to 11:59 AM. However, posting tweets during the hours of 12 PM to 5:59 PM, decreased the IRR for retweets by 40% (IRR 0.60, 95% CI 0.46-0.79, *P*<.001) compared with 6 AM to 11:59 AM. Posting on a Monday (IRR 3.57, 95% CI 2.50-5.09, *P*<.001) or Wednesday (IRR 1.50, 95% CI 1.11-1.11, *P*<.01) significantly increased the IRR compared with posting on a Thursday.

**Conclusions:**

Surveys are a useful tool to measure the knowledge, attitudes, and behaviors of a given population. Strategies to improve Twitter engagement include targeting low-profile accounts, posting tweets in the morning (12 AM-11:59 AM) or late evenings (6 PM-11:59 PM), and posting on Mondays and Wednesdays.

## Introduction

Recruiting participants and administering surveys for research are usually conducted in person, via post, or through email [1-3]. Recruiting participants and administering surveys using these methods can be expensive and time-consuming. A potential alternative to these traditional methods is the use of social media [4-6]. Social media is the virtual interaction among users that allows for the creation, sharing, and exchange of information via websites, such as Facebook, Twitter, and YouTube. As a recruitment tool, social media provides researchers with an efficient means to reach and engage with a large and diverse audience in a short period of time. Moreover, it is relatively low cost to administer and maintain.

An example of a social media platform that can be used for recruiting participants is the Web-based social networking site known as Twitter. Within a 140 character limit, short messages (tweets) can be posted with the inclusion of a link to a website, image, or video. Users can then share the tweet (retweet) with their virtual network or community (followers). This system of posting to one network of users, and reposting to a different network of users, enables researchers to recruit using the snowball sampling method [4,6]. Traditional snowball sampling techniques use social interaction between individuals, where a participant from within the target group will recruit other participants who share the same characteristics from their own network [6]. This technique is particularly important for recruiting hard-to-reach populations [7]. In sports, athletes and trainers that deal with concussion are not easy to access [8-12]. Recruiting via Twitter may therefore offer a useful research tool to reach and engage athletes and trainers that deal with concussion.

In addition to recruiting, Web-based surveys can be administered via Twitter by posting a unique link to a questionnaire. Although using Twitter to administer Web-based surveys for research is not new [4], strategies to improve response rates are yet to be reported in the literature. Depending on the purpose, a Twitter account or Twitter post can be designed to gain maximum and focused Web-based exposure, which potentially may increase response rates. For example, targeting specific accounts by mentioning them in the posts, or wording the post to persuade followers to ‘retweet’ or click on the survey link. Furthermore, the frequency of posts, time of day, day of the week, and whether to use more than one account to post tweets are factors that may affect the Twitter Web-based exposure and surveying response rates [13,14]. Yet, the best approach to use these Twitter features for research are currently unexplored.

Therefore, the purpose of this study was to compare the potential and actual reach of 2 Twitter accounts that administered a Web-based concussion survey to rugby union (henceforth, called ‘rugby’) players and trainers using 2 distinct twitter targeting strategies. Furthermore, determine the likelihood of receiving a retweet based on the time of the day and day of the week of posting the Web-based survey.

## Methods

### The Web-Based Survey

A survey based on previous concussion research was developed and exported to the Web-based survey website Survey Monkey. The survey comprised of 2 questionnaires, one for players, and one for those involved in the game (eg, coaches and athletic trainers). Survey Monkey allows for the routing of questions based on a participant’s response, and early on in the survey participants had to indicate whether they were a player or an individual involved in the game in some aspect other than playing. This response then automatically directed the participant onto the appropriate questionnaire. The survey commenced with the background to the study, and a space to provide informed consent. Thereafter, 9 general demographic questions were asked (gender, age, involvement in the sport, etc). Depending on whether the participant indicated they were a player or an individual involved in the game in some aspect other than playing, Survey Monkey directed the participant onto the relevant questionnaire. The player questionnaire consisted of 16 closed questions, with the other questionnaire comprising of 12 closed questions. Space was provided at the end of some questions to allow the participant to elaborate on their answer if they so desired. The final page allowed for further comment and feedback on the overall survey experience. All participants remained anonymous throughout the completion of the questionnaire. It was assumed that participants provided accurate and honest responses. The study was approved by the University of Cape Town Human Research Ethics Committee (HREC Ref: 210/2014).

### Social Media Strategy

After the link to the Web-based survey was available, the link to the survey was shared via Twitter. As no literature exists comparing different Twitter strategies to administer Web-based surveys for maximum and focused Web-based exposure, a decision was made to administer our concussion Web-based survey using 2 existing Twitter accounts, with each account executing a distinct targeting strategy. Using existing accounts also had the added benefit of having a starting base of followers. The personal accounts of the authors @Sharief_H and @SteveMellalieu were used. At the start of the study @Sharief_H had 1121 followers and @SteveMellalieu had 570 followers.

Next, a list of potential Twitter accounts to target was drawn up, and a list of predesigned tweets. The list contained International Rugby Organizations (eg, @IRBMedia, @IRBSevens), National Rugby Teams (eg, @WelshRugbyUnion, @EnglandRugby, @irfurugby), International Sport and Rugby Media (eg, @BBCSport, @BBCScrumV, @RugbyUnionNews), Professional Rugby Teams (eg, @Saracens, @CrusadersRugby), and High-Profile Professional Players (eg, @Rorylamont, @gareththomas14). The aforementioned accounts were considered ‘High-Profile’ because of their position to attract attention or publicity with a high social interaction potential. In contrast, ‘Low-Profile’ accounts belonged to University teams (eg, @ikeytigers, @BathUniRugby1, @cardiffmetrfc), nonprofessional rugby clubs (eg, @swansearfc, @carmquinsrfc), and rugby coaches, trainers, scientists, and administrators (eg, @timoconnorbl, @J_Darrall_Jones, @1RugbyCoach). Note, although in most cases the ‘High-Profile’ accounts had more than 10,000 followers, the number of followers an account had was not the main distinguishing criterion between ‘High-Profile’ and ‘Low-Profile’ accounts. The main distinguishing criterion was the position of the account to attract attention or publicity–‘High-Profile’ accounts were representative of professional players/international/national organizations, whereas ‘Low-Profile’ accounts were nonrepresentative accounts.

Tweets were designed to include who qualified to take part in the study, a request to complete the survey, the duration of the survey, the link to the survey, character space to mention an account(s), a request to retweet, and character space for hashtags. A hashtag (preceded by a # symbol that allows for users to search topics on Twitter) was created specifically for the study - #IRCR014 (International Rugby Concussion Research 2014), and other popular hashtags such #Rugby #Concussion were also used. Textbox 1 contains a list of tweets used.

**A list of tweets linked to the Web-based survey.**

*Pls RT Play/involved in rugby union? Pls take 2min 2 complete international concussion survey. [Link to survey]*

*Play or involved in rugby union as a player/coach/medical/admin? Pls take few min to complete online survey on concussion [Link to survey]*

*Player, Coach, Medical, Manager, in rugby union? Pls take 3-4min to complete online survey on concussion [Link to survey] Pls RT #rugby*

*If you play or are involved in rugby union, pls take part in an International Concussion study [Link to survey] Pls RT*

*@XXXXXX Pls RT Are you involved in rugby union as a player/coach, or medical personnel? Pls take few min to complete online survey on concussion experiences [Link to survey]*

Both accounts were at liberty to use any one of the above tweets. Administering the survey via Twitter commenced on April 2, 2014 and ended on August 3, 2014 (4 months). In the first 2 months of the study, the frequency of tweeting the survey was approximately 2 days of tweeting to targeted accounts, and then 2 days of no tweeting, alternating between the accounts. In the second 2 months, the posting occurred at least once a week to targeted accounts, alternating between the high *-* profile and low-profile strategy. Retweets from our own respective followers, and accounts not mentioned in the tweet post were also welcomed. For the majority of retweets received by targeted and nontargeted accounts, a reply tweet thanking the user was posted.

### Analysis of Tweets

Twitter exposure data for each account were extracted using a Web-based Twitter analytics software program called Twitonomy. Twitonomy provides data on the date and time of each tweet, the composition of the tweet in terms of text, whether the tweet was a new tweet or a retweet, the platform from where the tweet was sent, the number of retweets a tweet received, and the number of favorites a tweet received. All this data can be downloaded in a Microsoft Excel sheet, over a set period, with each row in the datasheet representing a tweet. Data were downloaded and analyzed for each account separately. In the downloaded excel sheet, tweets not pertaining to the study were deleted. In addition, only tweets with the link to the survey included and where users where targeted were extracted for analyses (ie, tweets thanking users or conversational tweets about the study were excluded from the analyses). The tweet data captured in the downloaded excel sheet were validated by comparing it with the actual posted tweets for each account. Thereafter, the user(s) mentioned in each tweet were identified, and the number of followers of that user was recorded. This represented the potential reach of the tweet. If the tweet contained more than one user, the number of followers for each account mentioned was added up, and the total number of followers represented the potential reach of that tweet. The total potential reach equaled the sum total of the potential reach of each tweet. For tweets with no accounts mentioned, potential reach was recorded from the number of followers of the account posting the tweet. The number of retweets received by each post represented the actual reach of the tweet–as retweeted posts meant that the followers of the targeted account(s) would actually see the post on their timeline feed. The number of retweets received by each account was further analyzed in order to understand when the most likely time of day and day of the week a retweet would be received. To analyze time, the 24 hours of the day were categorized into periods of 6 hours, specifically 12 AM to 5:59 AM, 6 AM to 11:59 AM, 12 PM to 5:59 PM, and 6 PM to 11:59 PM. The date of the tweet was used to determine the day of the week.

Descriptive statistics are reported to compare potential reach, actual reach, and time and day of the week ‘retweets’ were received between the high-profile and low-profile strategy. In addition, a Student *t* test was used to compare potential reach and actual reach between the high-profile and low-profile strategy. Statistical significance was set at *P*<.05. For *t* test comparisons, the mean number of followers and standard deviations for each strategy are reported. Cohen’s *d* effect sizes were calculated to determine the magnitude of the difference between the two strategies. Effect sizes of <0.2, 0.2-0.6, 0.6-1.2, and 1.2-2 were considered trivial, small, moderate, and large, respectively [15].

To determine the best strategy, and the most likely time of day and day of the week a retweet was received based on the number of posts, Poisson regression analyses was used. Poisson regression allows for the determination of the relationship between predictor variables (in this case, strategy, time of day, and day of the week) and the counts of events (outcome variable) while taking into account exposure (number of posted tweets). The outcome variable for this Poisson regression was number of retweets received. First, a Poisson regression model was performed for each predictor variable separately (ie, one for strategy, time of day, and day of the week). Thereafter, a model with time of day and day of the week as the predictor variables was adopted. The final model contained all three predictor variables in one model. To perform this analysis, predictor variables were computed relative to a referent or base variable. For strategy, the referent variable was the low-profile strategy; for time of day, the referent variable was 6 AM to 11:59 AM; and for day of the week, the referent variable was Thursday. Incidence rate ratio (IRR) and 95% confidence intervals (CI) were reported for each predictor variable. The standard interpretation for the Poisson regression model is that for a unit change in the predictor variable (relative to its referent variable), the incidence rate is expected to change by its respective parameter estimate (IRR), while holding all other variables in the model constant.

## Results

### Descriptive

**Figure 1:**
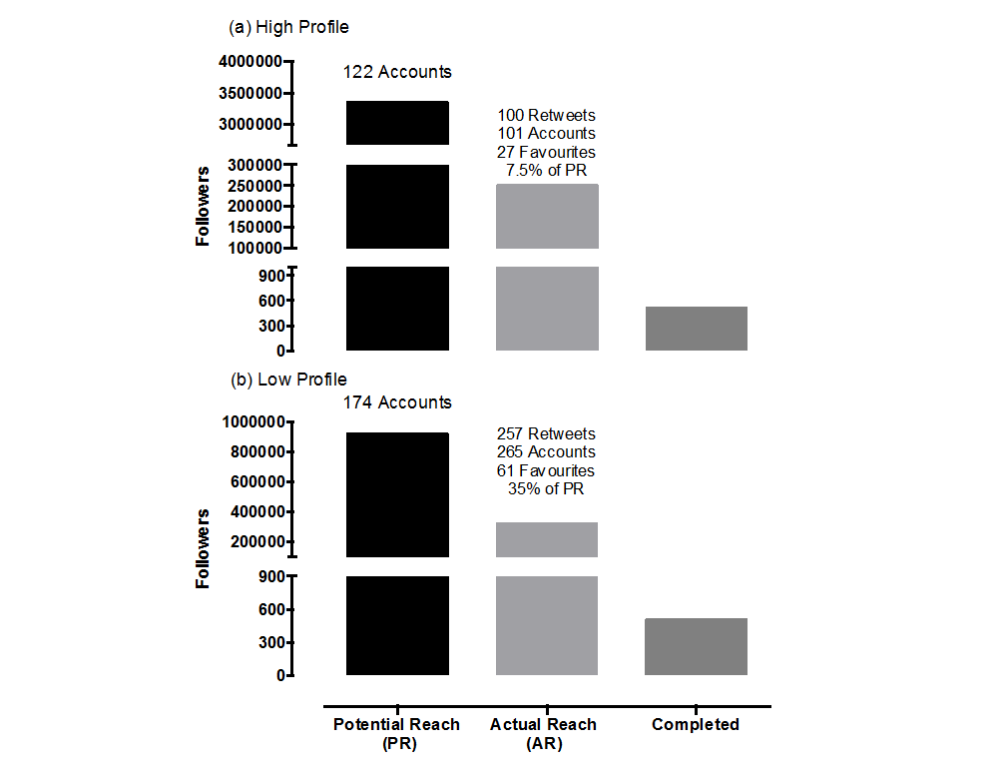
The number of followers high profile and low profile accounts could potentially have reached, actually reached, and the number of followers that completed Web-based questionnaires.

Over 4 months, 507 questionnaires were completed. The primary involvements of the respondents are reported in Table 1. The high-profile strategy tweeted 146 posts (including ‘thank you for retweets’) and mentioned 122 accounts with a potential reach of 3,352,223 followers. The high profile strategy received 100 retweets from 101 accounts, which totaled an actual reach of 249,836 followers (Figure 1). This represented 7.5% (n=249,836) of the potential reach. Of the high-profile tweets, 27 were saved as favorites. In comparison, the low-profile strategy tweeted 164 posts (including ‘thank you for retweets’) and mentioned 174 accounts with a potential reach of 921,421 followers. The low *-* profile strategy received 257 retweets from 265 accounts, which totaled to an actual reach of 323,796 followers. This represented 35% of the potential reach. Of the low-profile strategy, 61 tweets were favorited. The high-profile strategy had a higher potential reach than the low-profile strategy (difference 2,430,802 followers), but the low-profile strategy had a higher actual reach (difference 73,960 followers).

### High-Profile Strategy Versus Low-Profile Strategy

The potential reach of the high-profile strategy (27477±64819) was significantly different (*P*<.001) to the low-profile strategy (5295±9979), although the magnitude of this difference was small (effect size=0.52). On average, the actual reach of the high-profile strategy (2473±6686) was significantly different (*P*<.01) to the low-profile strategy (1221±2424). The magnitude of this difference however, was small (effect size=0.31).

**Table 1:** The primary involvements of the respondents to the Web-based survey (n=472).

Primary Involvement^a^	%	n
Player	46%	219
Former player (retired)	13%	59
Coach	13%	62
Referee/official	4%	19
Administrator	2%	8
Team manager	2%	9
Physiotherapist/athletic trainer	6%	30
Sports physician	1%	5
General practitioner	0%	1
Support staff (sports scientist, S&C coach, nutritionist, psychologist, analyst)	4%	18
Parent	3%	12
Other	6%	30

^a^35 respondents did not answer this question.

### Poisson Regression

#### Predictor Variables Modelled Independently From Each Other

The number of retweets received by a Twitter account decreased by 56% when using the ‘high- profile strategy’ compared with the ‘low-profile strategy’ (IRR 0.44, 95% Cl 0.35-0.55, *P*<.001). For time of day, the number retweets received during 12 AM to 5:59 AM (IRR 1.08, 95% Cl 0.74-1.57, *P*>.05), 6 PM to 11:59 AM (IRR 1.03, 95% Cl 0.75-1.41, *P*>.05) did not significantly change compared with 6 AM to 11:59 AM. However, posting tweets during 12 PM to 5:59 PM, decreased the IRR for retweets by 34% (IRR 0.66, 95% Cl 0.51-0.85, *P*=.001) compared with 6 PM to 11:59 AM. The IRR was 3.30 (95% Cl 2.33-4.66, *P*<.001), and 1.48 (95% Cl 1.10-1.99, *P*<.01) times higher for retweets when posting on a Monday and Wednesday, respectively.

#### Time of Day and Day of the Week Model

When time of day and day of the week were factored into one model, posting tweets during 12 PM to 5:59 PM, decreased the IRR for retweets by 23% (IRR 0.77, 95% Cl 0.59-0.99, *P*<.05) compared with 6 AM to 11:59 AM. Monday (IRR 3.24, 95% Cl 2.27-4.62, *P*<.001) and Wednesday (IRR 1.50, 95% Cl 1.12-2.03, *P*<.01) remained the best days to post tweets.

#### Strategy, Time of Day, and Day of the Week Model

In the full model, the number of retweets received by a Twitter account decreased by 72% when using the ‘high-profile strategy’ compared with the ‘low-profile strategy’ (IRR 0.28, 95% Cl 0.21-0.37, *P*<.001). When taking into account strategy and day of the week, the IRR for the number retweets received during 12 AM to 5:59 AM (IRR 2.98, 95% Cl 1.88-4.71, *P*>.001) and 6 PM to 11:59 PM (IRR 1.48, 95% Cl 1.05-2.09, *P*>.05) increased relative to 6 AM to 11:59 AM. However, posting tweets during 12 PM to 5:59 PM, decreased the IRR for retweets by 40% (IRR 0.60, 95% Cl 0.46-0.79, *P*<.001) compared with 6 AM to 11:59 AM. Posting on Monday (IRR 3.57, 95% Cl 2.50-5.09, *P*<.001) or Wednesday (IRR 1.50, 95% Cl 1.11-1.11, *P*<.01) significantly increased the IRR compared with Thursday.

## Discussion

### Principal Results

The study yielded 4 main results: (1) this is the first study comparing the Web-based exposure of two Twitter strategies for Web-based surveying, (2) the low-profile strategy was more likely to receive retweets when the number of tweets was taken into account, (3) the time of day with the least potential to elicit retweets was between 12 PM and 5:59 PM, and (4) the day of the week with the highest potential to elicit retweets was a Monday and Wednesday.

### High-Profile Strategy Versus Low-Profile Strategy

This is the first study comparing the social media exposure of two Twitter strategies for Web-based surveying. Twitter strategies for this study were based on the accounts each strategy targeted in a tweet. Although the high-profile strategy had a significantly higher mean potential and actual reach than the low-profile strategy, the magnitude of this difference was small. Furthermore, the low-profile strategy was more likely to receive retweets when the number of tweets was taken into account. High-profile Twitter accounts that are representative of a professional organization may be governed by the rules of the organization, and therefore less inclined to engage and retweet posts they are mentioned in. In contrast, low-profile Twitter accounts may be more liberal in their Twitter engagement, and willing to share tweets for research purposes.

### Time of Day and Day of the Week

The time of day with the least potential to elicit retweets was between 12 PM and 5:59 PM, and days with the highest potential to elicit retweets were a Monday and Wednesday. Interestingly, the time of day and the day of the week with the highest potential to elicit retweets in this study differ to the recommendations offered for business marketing. According to Zarrella [14], retweets for business marketing purposes are highest for tweets posted between 3 PM and 5 PM and on Thursdays and Fridays [14]. Although the results show that the potential to receive a retweet may differ throughout the day and on different days of the week, it should be acknowledged that Twitter has a global community across all time zones, and the dynamic and emerging nature of posting tweets and retweeting makes it difficult to recommend an exact time and day to post a Web-based survey. With that said, because this is the first study to explore the relationship between time and day of posting a Web-based survey on Twitter and its potential to elicit a retweet, the results reported here can be used as a guideline.

### Determinants of Retweets

Retweeting, arguably, is the most important feature of Twitter, as this allows for the propagation of information. Retweeting is considered a behavior of selecting and diffusing information [16]. In view of this, a number of studies have been conducted to determine why Twitter users retweet certain posts compared with others [16-18]. Based on this work, determinants of retweeting can be divided into two categories–content and contextual [18]. In brief, content features relate to the composition of the tweet–whether the information in the tweet is positive, objective, or contains a verb or not. For example, Suh et al [18] showed that adding a link or hashtag to a tweet increases the probability of a retweet. Contextual features relate to the number of followers a user has, the trustworthiness of the user, the expertise of the user, and knowing information about one’s followers. For example, Rudat et al [16] showed that users that knew more about their followers adapted their retweets to serve the needs or expectations of their followers. In light of the above literature, and considering both accounts in this study used similar content, some contextual differences between the 2 accounts used is this study may explain why the low-profile strategy was more likely to receive retweets.

Although personal accounts were used for posting tweets and administering the survey, the name of the account that executed the low-profile strategy is called ‘Rugby Science’, whereas the name of the account that executed the high-profile strategy was ‘Stephen Mellalieu’–the author’s name. Contextual features such as trustworthiness and expertise, based on the name of the account ‘Rugby Science’ may have influenced retweeting behavior, as this was a Rugby study. This highlights a caveat in the current study, but may offer a recommendation for future Web-based surveying (ie, align the name of the account posting the survey, to the area of study).

### Limitations

Even though the objective of this study was achieved, we were unable to determine the relationship between the Twitter strategy used, and the number of completed questionnaires. The Twitter analytics software program used in this study was sufficient to analyze reach and retweet data. Presently, Twitter offers its own analytical services that allows for the analyses of the number of users that viewed and engaged with each tweet, and the number of users that click the link posted in a tweet (if a link is provided). Even though this will provide data on the number of users that clicked on the link to the survey, whether the user completed the questionnaire cannot be determined. For future work in this area, a potential solution to this limitation would be to add a question to the questionnaire, asking from which Twitter account was the link to the survey accessed. A final noteworthy limitation of this study is that the identity of each participant could not be verified. To address this limitation, contact details of the participant’s current club or team should be obtained in the survey, and subsequently contacted to verify their identity.

### Conclusion

Surveys are a useful tool to measure the knowledge, attitudes, and behaviors of a given population. As a recruitment tool, social media provides researchers with an efficient means to reach and engage with a large and diverse audience in a short period of time. Moreover, it is relatively low cost to administer and maintain. Twitter allows for the virtual social interaction among a network of users that enables researchers to recruit and administer Web-based surveys using the snowball sampling method. Strategies to improve Twitter engagement include, targeting low-profile accounts, posting tweets in the morning (12 AM-11:59 AM) or late evenings (6 PM-11:59 PM), and posting on Mondays and Wednesdays.

## Abbreviations

CI: confidence interval
IRR: incidence rate ratio

## References

[1] Edwards PJ, Roberts I, Clarke MJ, Diguiseppi C, Wentz R, Kwan I, Cooper R, Felix LM, Pratap S (2009). Methods to increase response to postal and electronic questionnaires. Cochrane Database Syst Rev.

[2] Treweek S, Lockhart P, Pitkethly M, Cook JA, Kjeldstrøm M, Johansen M, Taskila TK, Sullivan FM, Wilson S, Jackson C, Jones R, Mitchell ED (2013). Methods to improve recruitment to randomised controlled trials: Cochrane systematic review and meta-analysis. BMJ Open.

[3] Pit SW, Vo T, Pyakurel S (2014). The effectiveness of recruitment strategies on general practitioner's survey response rates - a systematic review. BMC Med Res Methodol.

[4] O'Connor A, Jackson L, Goldsmith L, Skirton H (2014). Can I get a retweet please? Health research recruitment and the Twittersphere. J Adv Nurs.

[5] Quach S, Pereira JA, Russell ML, Wormsbecker AE, Ramsay H, Crowe L, Quan SD, Kwong J (2013). The good, bad, and ugly of online recruitment of parents for health-related focus groups: lessons learned. J Med Internet Res.

[6] Close S, Smaldone A, Fennoy I, Reame N, Grey M (2013). Using information technology and social networking for recruitment of research participants: experience from an exploratory study of pediatric Klinefelter syndrome. J Med Internet Res.

[7] Sadler GR, Lee H, Lim RS, Fullerton J (2010). Recruitment of hard-to-reach population subgroups via adaptations of the snowball sampling strategy. Nurs Health Sci.

[8] Clay MB, Glover KL, Lowe DT (2013). Epidemiology of concussion in sport: a literature review. J Chiropr Med.

[9] Noble JM, Hesdorffer DC (2013). Sport-related concussions: a review of epidemiology, challenges in diagnosis, and potential risk factors. Neuropsychol Rev.

[10] Sullivan SJ, Schneiders AG, Cheang C, Kitto E, Lee H, Redhead J, Ward S, Ahmed OH, McCrory PR (2012). 'What's happening?' A content analysis of concussion-related traffic on Twitter. Br J Sports Med.

[11] Williams D, Sullivan SJ, Schneiders AG, Ahmed OH, Lee H, Balasundaram AP, McCrory PR (2014). Big hits on the small screen: an evaluation of concussion-related videos on YouTube. Br J Sports Med.

[12] Khurana VG, Kaye AH (2012). An overview of concussion in sport. J Clin Neurosci.

[13] Cooper BB (2013). A Scientific Guide to Posting Tweets, Facebook Posts, Emails and Blog Posts at the Best Time.

[14] Zarrella D (2013). The Science of Marketing: When to Tweet, what to Post, how to Blog, and Other Proven Strategies.

[15] Hopkins WG, Marshall SW, Batterham AM, Hanin J (2009). Progressive statistics for studies in sports medicine and exercise science. Med Sci Sports Exerc.

[16] Rudat A, Buder J, Hesse FW (2014). Audience design in Twitter: retweeting behavior between informational value and followers’ interests. Computers in Human Behavior.

[17] Liu Z, Liu L, Li H (2012). Determinants of information retweeting in microblogging. Internet Research.

[18] Suh B, Hong L, Pirolli P, Chi E (2010). Want to be retweeted? Large scale analytics on factors impacting retweet in twitter network. Social computing (socialcom).

